# Pituitary Metastasis of Lung Neuroendocrine Carcinoma Mimicking Pituitary Adenoma:Case Report and Literature Review

**DOI:** 10.3389/fendo.2021.678947

**Published:** 2021-07-14

**Authors:** Xiaohai Liu, Renzhi Wang, Mingchu Li, Ge Chen

**Affiliations:** ^1^ Department of Neurosurgery, Xuanwu Hospital Capital Medical University, Beijing, China; ^2^ Chinese Pituitary Specialists Congress, Beijing, China; ^3^ Department of Neurosurgery, Peking Union Medical College Hospital, Chinese Academy of Medical Sciences and Peking Union Medical College, Beijing, China

**Keywords:** pituitary metastasis, lung neuroendocrine carcinoma, pituitary adenoma, case report, diabetes insipidus

## Abstract

Pituitary metastasis is an unusual situation in clinical practice, while the incidence is increasing with age. Breast cancer for women and lung cancer for men were the most frequent primary origins of pituitary metastasis. Diagnosing asymptomatic patients with unknown primary malignant origin is difficult, thus pituitary metastasis may be diagnosed as primary pituitary adenoma. Here, we report a case of a 65-year-old patient with visual changes and diabetes insipidus, showing an extensive mass in the sellar region which was initially thought to be a primary pituitary adenoma. Patient corticotropic deficits were corrected, and transnasal transsphenoidal surgery was adopted, leading to total tumor resection. Tumor texture during surgical procedure was similar to that of pituitary adenoma. However, the histopathological and immunohistochemistry results suggested it as a pituitary metastasis from lung neuroendocrine tumor. Postoperative chest CT scan confirmed a pulmonary mass consistent with primary neoplasm. Abdominal CT further detected multiple metastases in liver, pancreas, and colon. Despite intensive treatment, the patient continued to show decreased level of consciousness due to cachexia, resulting in death 1 week after surgery. This case highlights the importance of differential diagnosis of invasive lesions of the sellar region, especially in individuals over 60 years of age with diabetes insipidus.

## Introduction

The incidence of pituitary metastasis is very low, accounting for approximately 1% of all intracranial metastases (1). As clinical symptoms of pituitary metastasis are similar to those of other sellar tumors and Magnetic resonance imaging (MRI) is nonspecific, pituitary metastases are easily misdiagnosed. Here, we report a 65-year-old patient harboring pituitary metastasis derived from lung neuroendocrine carcinoma, was misdiagnosed and died of cachexia after surgery. We hope our case report will highlight the importance of differential diagnosis for invasive lesions of the sellar region, especially in individuals over 60 years of age with diabetes insipidus. It is possible that invasive pituitary lesions could be metastasized from unknown primary neoplasm, although the incidence is extremely low.

## Case Presentation

**Figure 1:**
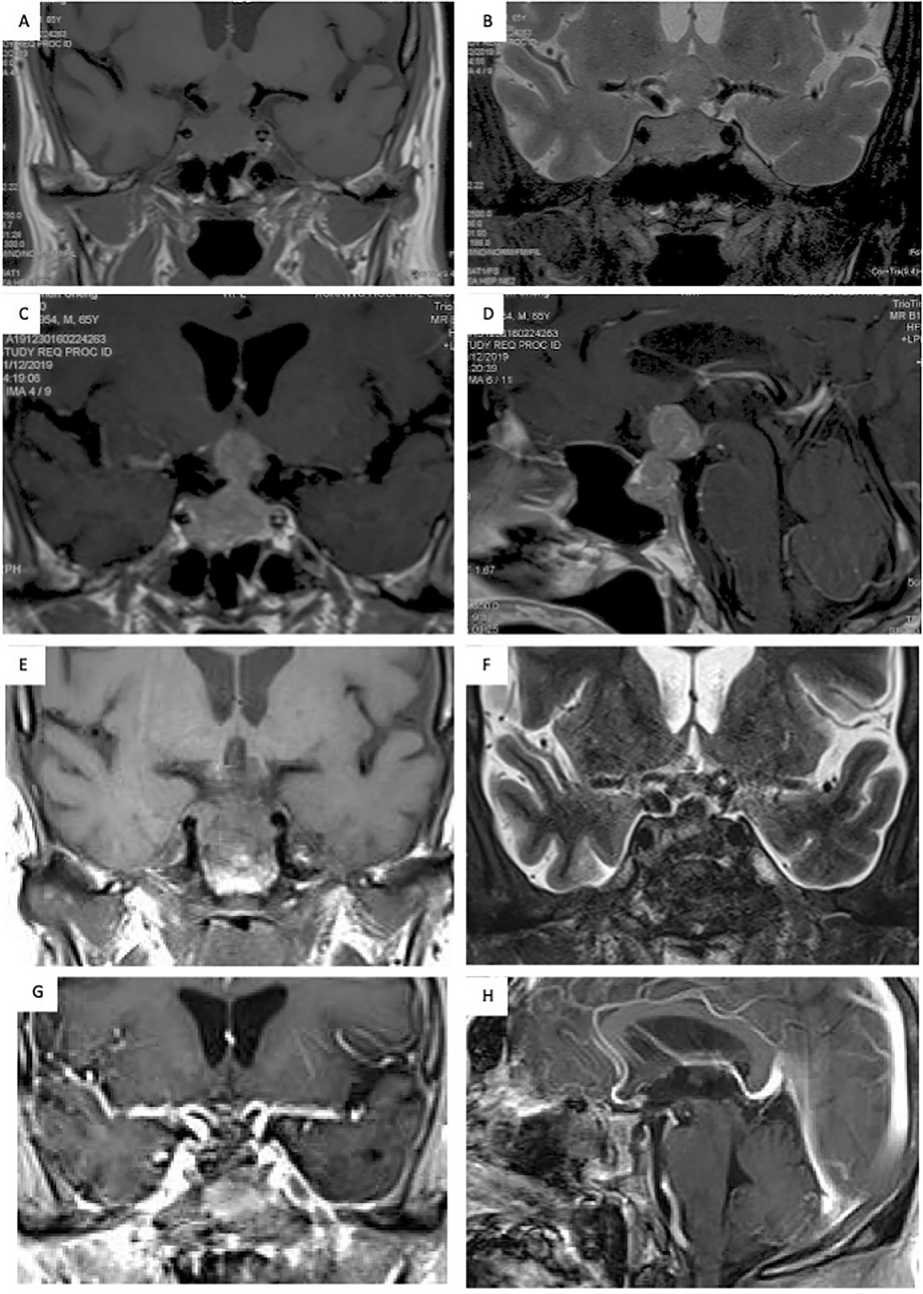
Seller MRI scans showed a sellar lesion with suprasellar extension and compression of the optic chiasm. **(A, B)** The lesion located in the sellar region presented with an isointense signal on T1- and T2-weighted MRI; **(C, D)** The mass was uniformly enhanced on MRI after contrast enhancement, and a pituitary adenoma was highly suspected. **(E–H)** The postoperative MRI showed total resection of the lesion.

The patient was a 65-year-old male who presented with progressive impaired vision and diabetes insipidus (DI) for 2 months. Visual fields of both eyes were relatively normal, and no past cancer history was reported. MRI indicated a sellar lesion with isointense signal on T1- and T2-weighted images which was homogenously enhanced after contrast MRI. The lesion was approximately 36 mm × 24 mm × 17 mm (Knosp grade 3) with suprasellar extension and compression of the optic chiasm. A pituitary adenoma was highly suspected (**Figures 1A–D**). Hormonal evaluation revealed hypoadrenocorticism and hypogonadism (**Table 1**). Other routine laboratory tests did not show abnormality.

**Table 1:** Initial hormonal evaluation, indicating hypoadrenocorticism and hypogonadism of the patient.

Hormone	Result	Reference
Testosterone (ng/ml)	26.93	175.0-781.0
PRL (ng/ml)	6.55	0-22.0
TSH (uIU/ml)	0.05	0.34-5.6
FT4 (ng/dl)	0.92	0.89-1.76
Cortisol (ug/dl)	1.88	5.0-25.0
ACTH (pg/ml)	5.13	7.2-63.3
IGF-1 (ng/ml)	25	75-212

**Figure 2:**
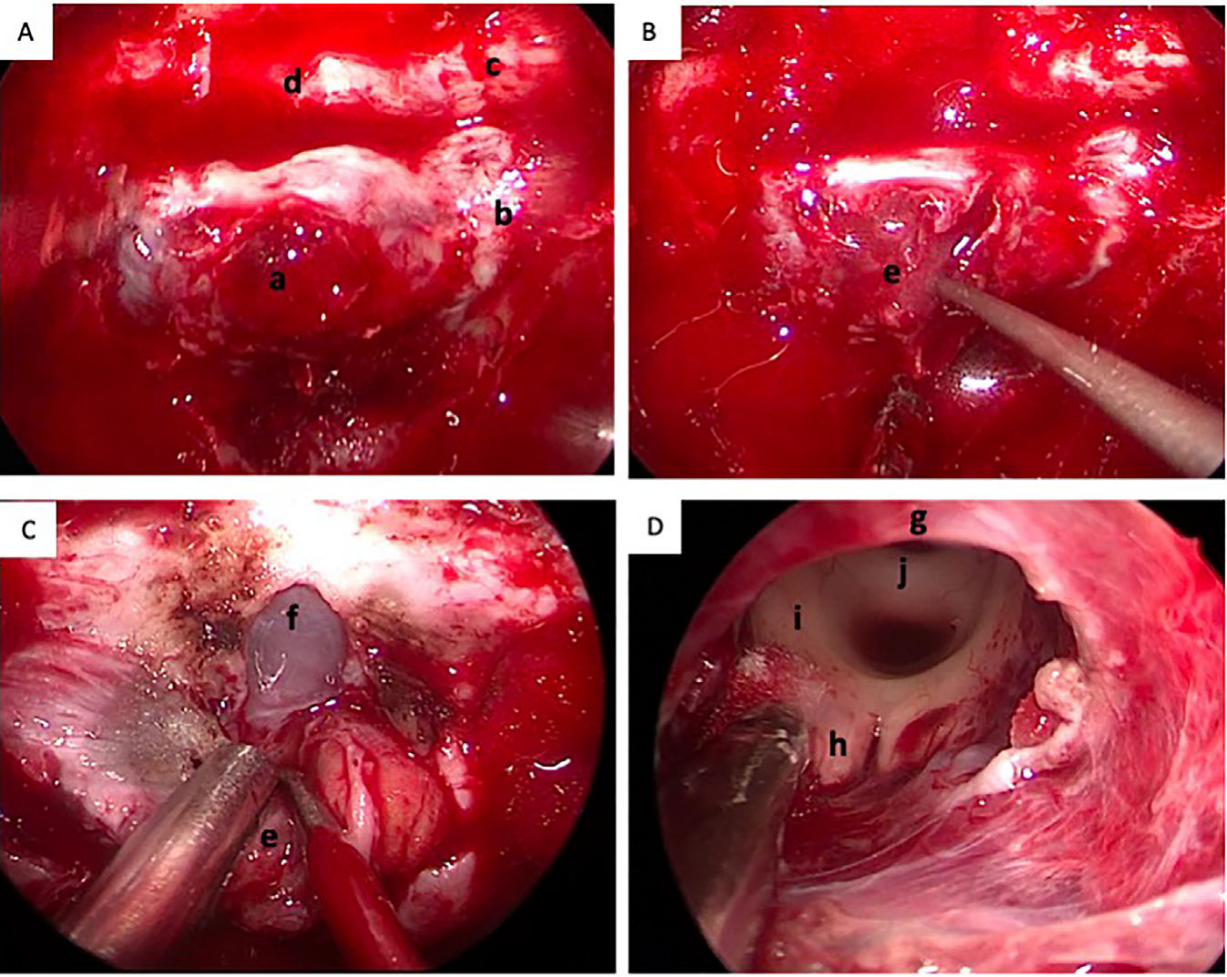
Intra-operative conditions of the lesion. **(A)** The dura of sellar floor was invaded by the tumor; **(B)** The lesion was soft, mimicking pituitary adenoma in texture (the arrow); **(C)** The dura of sphenoid platform was opened; **(D)** The lesion was totally resected and the third ventricle was revealed. a, the invaded dura of the sellar floor; b, the cavernous sinus; c, the optic nerve; d, the sphenoid platform; e, the tumor; f, the arachnoid membrane of sphenoid platform; g, the optic chiasm; h, the mamillary body; i, posterior commissure.

Subsequently, the patient underwent an endoscopic transsphenoidal surgery. During the surgical operation, the lesion was found to be soft, mimicking pituitary adenoma in texture (**Figures 2A–C**). The lesion was totally resected (**Figure 2D**). Postoperatively, the patient’s impaired vision was improved, but diabetes insipidus persisted. And the postoperative MRI showed total resection of the lesion (**Figures 1E–H**).

## Postoperative Treatment

**Figure 3:**
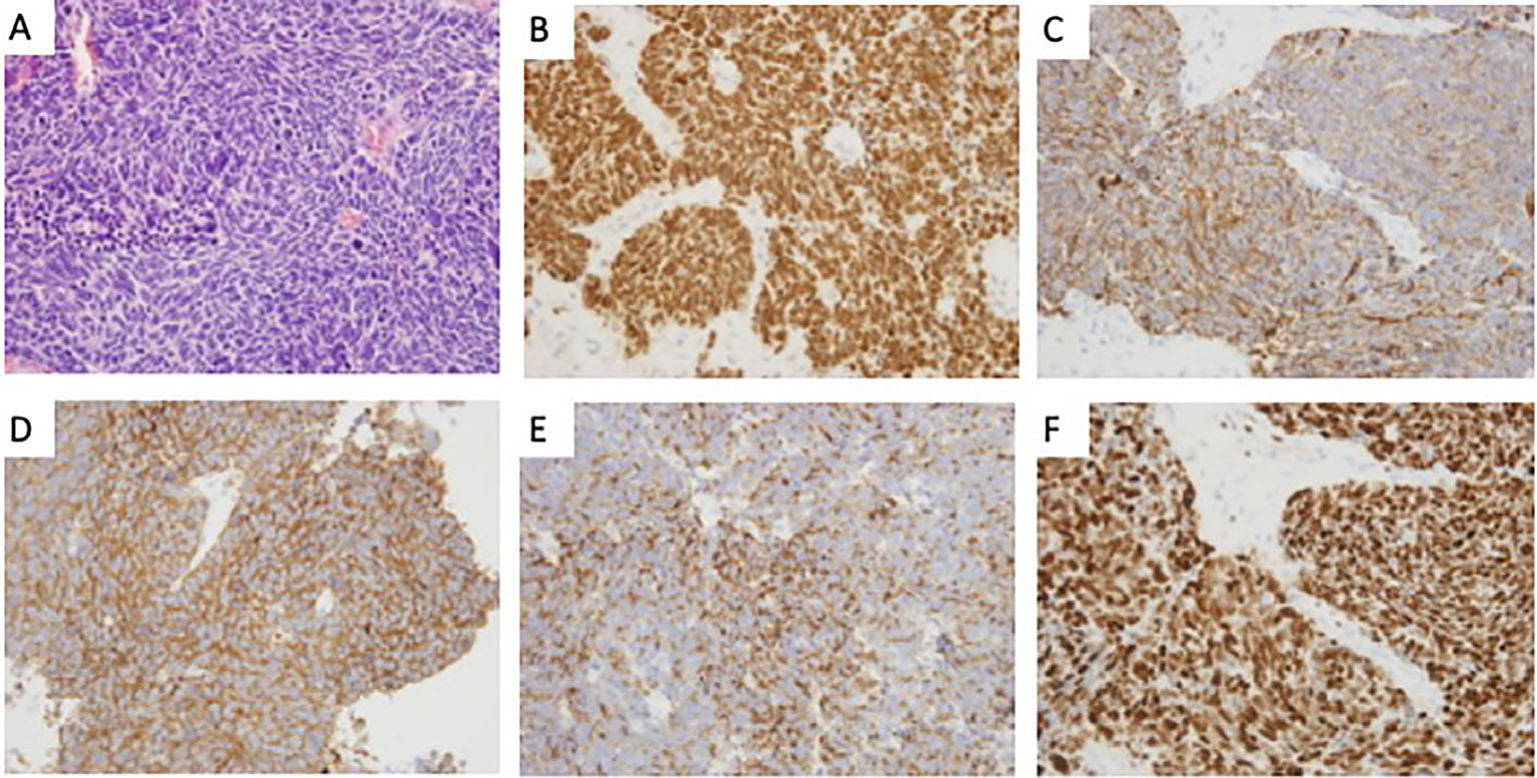
The histological features of the lesion revealed pituitary metastasis of lung neuroendocrine. **(A)** Hematoxylin and eosin (H&E) staining revealed a solid tumor involving the pituitary gland, characterized by small cells with high-grade nuclear atypia and fibrosis (×400); **(B–E)** Immunohistochemistry revealed positivity for TTF-1(B), CK(C), Syn(D), and CgA(E) (×400); **(F)** Immunohistochemical staining of Ki-67 (×400).

Hematoxylin and eosin (H&E) staining showed a solid tumor involving the pituitary gland, and characterized by small cells with high-grade nuclear atypia and fibrosis (**Figure 3A**). Immunohistochemistry revealed positivity for CK (**Figure 3B**), TTF-1 (**Figure 3C**), Syn (**Figure 3D**), and CgA (**Figure 3E**) and negativity for vimentin and LCA. The Ki-67 index was 90%, indicating a neuroendocrine carcinoma (**Figure 3F**). Postoperative chest CT scan confirmed a pulmonary mass consistent with the primary neoplasm and abdominal CT indicated multiple metastases in liver, pancreas, and colon. Although immunostain for all these markers may be positive in primary pituitary neuroendocrine carcinoma, pituitary metastasis from lung neuroendocrine carcinoma is far more likely than a primary pituitary neuroendocrine carcinoma with liver and lung metastasis. Despite intensive treatment, the patient continued to show decreased level of consciousness due to cachexia, resulting in death 1 week after surgery.

## Discussion

Pituitary adenoma is the most common sellar lesion, constituting approximately 15% of all intracranial neoplasms (2), while pituitary metastasis accounts for only approximately 1% of all pituitary adenomas in previous serial studies (1). With the aging population, the occurrence of pituitary metastasis is increasing in clinical practice. Tumors originating from multiple organs can metastasize to the sellar region, such as liver cancer, renal cell carcinoma, thyroid cancer, and prostate cancer, among which the most common primary malignancies are breast cancer in female and lung cancer in male, and these two cancers account for approximately two-thirds of all cases (3).

It is often difficult to distinguish pituitary metastasis from primary pituitary adenomas, as the clinical and radiological features of pituitary metastases are usually nonspecific (4). Most pituitary metastases are clinically silent, and only 6.8% of cases are symptomatic (5). The most common clinical manifestations include DI, visual field defects and hypopituitarism. As DI is very race and occurs only in less than 1% of patients with pituitary adenomas, the possibility of pituitary metastasis could be increased in patients with DI (6). According to Siqueira, et al., it could be a differential diagnosis of invasive lesions in the sellar region, mainly in patients over 50 years of age and/or associated with diabetes insipidus. Therefore, the presence of DI is a useful distinguishing factor, which is more common in pituitary metastasis than in PA. Radiological findings of pituitary metastasis are not specific (7), although some authors mentioned that the most typical radiographic features of metastatic pituitary tumors were enlargement or enhancement of the pituitary stalk with a pituitary mass (8). In our case, the patient presented with clinical visual changes and diabetes insipidus, which is not common in PA. However, the radiological findings of the patient showed typical snowman-shaped appearance of a pituitary adenoma. As there was no previous history of malignancy reported, clinical suspicion of pituitary metastases was low, and diagnosis of nonfunctional PA was made.

Neuroendocrine carcinomas metastasizing to the pituitary gland are extremely rare (9). In a recent review, 15 cases of pituitary metastases were reported, most of which were from neuroendocrine carcinomas (10). The treatment of pituitary metastasis requires to be individualized. Appropriate treatment should be taken based on the patient’s primary tumor situation, clinical symptoms, and physical conditions (11). The main therapeutic methods include surgery, radiotherapy and chemotherapy. Patient survival was generally determined by the type of primary tumor, while the overall prognosis was poor, with a median survival of only 12.9 months (12, 13). Surgical treatment is typically suitable for symptomatic patients with pituitary metastasis, as this approach is designed for symptom relief and provides a pathological diagnosis for subsequent treatment. Unfortunately, surgery cannot extend the overall survival of patients (14, 15). Although tumor resection of pituitary metastasis by transsphenoidal surgery or craniotomy is safe and effective, pituitary metastasis, which is highly aggressive, often destroys the dura of the sellar base and diaphragm; tough texture of pituitary metastases, tight adhesions to surrounding tissues, and abundant blood supply make it difficult to achieve total resection (15). Therefore, systemic chemotherapy and targeted therapy should be adopted according to the type of primary tumor, which is the most important factor affecting the overall prognosis and progression-free survival of patients (16). In our case, despite intensive treatment, the patient continued to show a decreased level of consciousness due to cachexia, resulting in death one week after surgery.

A small but accumulating numbers of literature described clinical and histopathological correlations with pituitary metastases derived from neuroendocrine tumors, however, genetic basis underlying this presentation remains poorly characterized. Christopher, et al. reported a case of a 68-year-old with a history of lung carcinoid tumor who developed a suprasellar lesion, in which key mutations in PTCH1 and BCOR that have been previously implicated in both systemic neuroendocrine and primary pituitary tumors with potentially actionable therapeutic targets (17).

## Conclusion

Although the incidence of pituitary metastasis is very low and its clinical symptoms and MRI findings are similar to those of other sellar tumors, its progression is fast and prognosis is poor. Our case highlights the importance of a differential diagnosis of invasive lesions of the sellar region, mainly in individuals over 60 years of age with diabetes insipidus.

## Data Availability Statement

The raw data supporting the conclusions of this article will be made available by the authors, without undue reservation.

## Ethics Statement

The studies involving human participants were reviewed and approved by Research Ethics Committee of Xuanwu Hospital. The patients/participants provided their written informed consent to participate in this study. Written informed consent was obtained from the individual(s) for the publication of any potentially identifiable images or data included in this article.

## Author Contributions

All four authors were involved in patient treatment, data collection and analysis, and manuscript writing. All authors contributed to the article and approved the submitted version.

## Funding

Financial support for this study was provided by the Scientific Research Project of Capital Health Development in 2018 (grant number: 2018-4-4018). The funding institutions played no role in the design of the study, data collection or analysis, decision to publish, or preparation of the manuscript.

## Conflict of Interest

The authors declare that the research was conducted in the absence of any commercial or financial relationships that could be construed as a potential conflict of interest.

## Abbreviations

ACTH, adrenocorticotropic hormone; CgA, chromogranin; CK, cytokeratin; CT, computed tomography; DI, diabetes insipidus; H&E, Hematoxylin and eosin; IGF-1, insulin like growth factor-1; LCA, leukocyte common antigen; MRI, magnetic resonance imaging; PRL, prolactin; T4, thyroxine; Syn, synaptophysin; TSH, thyroid stimulating hormone; TSS, trans-sphenoidal surgery; TTF1, thyroid transcription factor 1.
